# Functional genomics in Spiralia

**DOI:** 10.1093/bfgp/elad036

**Published:** 2023-08-09

**Authors:** Francisco M Martín-Zamora, Billie E Davies, Rory D Donnellan, Kero Guynes, José M Martín-Durán

**Affiliations:** School of Biological and Behavioural Sciences, Queen Mary University of London, Mile End Road, London E1 4NS, UK; School of Biological and Behavioural Sciences, Queen Mary University of London, Mile End Road, London E1 4NS, UK; School of Biological and Behavioural Sciences, Queen Mary University of London, Mile End Road, London E1 4NS, UK; School of Biological and Behavioural Sciences, Queen Mary University of London, Mile End Road, London E1 4NS, UK; School of Biological and Behavioural Sciences, Queen Mary University of London, Mile End Road, London E1 4NS, UK

**Keywords:** Spiralia, genomics, epigenomics, transcriptomics, spiral cleavage, evolution

## Abstract

Our understanding of the mechanisms that modulate gene expression in animals is strongly biased by studying a handful of model species that mainly belong to three groups: Insecta, Nematoda and Vertebrata. However, over half of the animal phyla belong to Spiralia, a morphologically and ecologically diverse animal clade with many species of economic and biomedical importance. Therefore, investigating genome regulation in this group is central to uncovering ancestral and derived features in genome functioning in animals, which can also be of significant societal impact. Here, we focus on five aspects of gene expression regulation to review our current knowledge of functional genomics in Spiralia. Although some fields, such as single-cell transcriptomics, are becoming more common, the study of chromatin accessibility, DNA methylation, histone post-translational modifications and genome architecture are still in their infancy. Recent efforts to generate chromosome-scale reference genome assemblies for greater species diversity and optimise state-of-the-art approaches for emerging spiralian research systems will address the existing knowledge gaps in functional genomics in this animal group.

## INTRODUCTION

**Figure 1:**
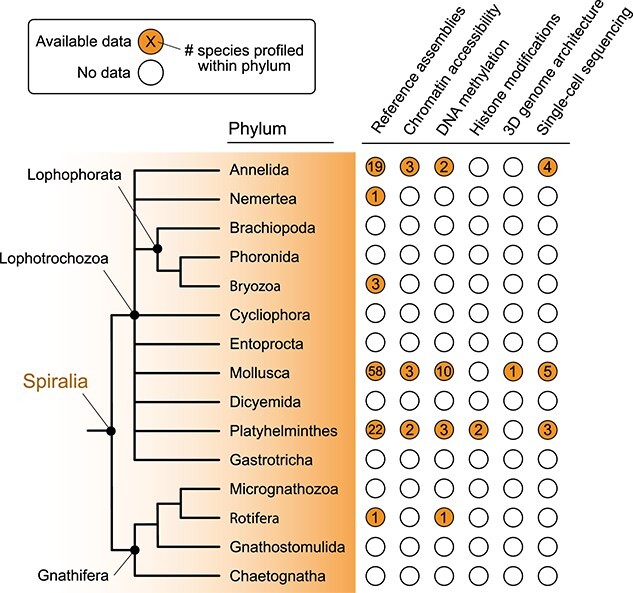
State-of-the-art spiralian functional genomics. Cladogram depicting the evolutionary relationships of all 15 spiralian phyla. Next to the topology, for each phylum, we show the number of species with reference (chromosome-scale) genome assemblies (as per the NCBI Genome database) and the presence or absence of functional genomics data of each of the five aspects of gene expression regulation we have reviewed: chromatin accessibility, DNA methylation, histone modifications, 3D genome architecture and single-cell transcriptomic sequencing. Numbers inside the orange circles depict the number of species within that phylum with available data for each aspect of genome regulation.

Spiralia is, together with Ecdysozoa (e.g. insects and nematodes) and Deuterostomia (e.g. echinoderms and vertebrates), one of the three major lineages of bilaterally symmetrical animals [1, 2]. Although it includes over half of the animal phyla and many economically and biomedically important invertebrate species, like bivalves, octopuses, earthworms and parasitic flatworms, the study of Spiralia is, compared with other animal lineages, often overlooked. This is particularly relevant in functional genomics and the analysis of gene expression regulation, where the lack of available high-quality genome assemblies, optimised protocols and critical mass in the research community has hindered our understanding of the genome regulatory mechanisms sustaining this large fraction of the animal diversity (Figure 1). In this manuscript, we focus on five aspects of genome regulation—chromatin accessibility, DNA methylation, histone post-translational modifications, genome architecture and single-cell transcriptomics—to review our current understanding of this process in Spiralia and propose future steps to address the existing limitations and open questions.

## CHROMATIN ACCESSIBILITY

**Figure 2:**
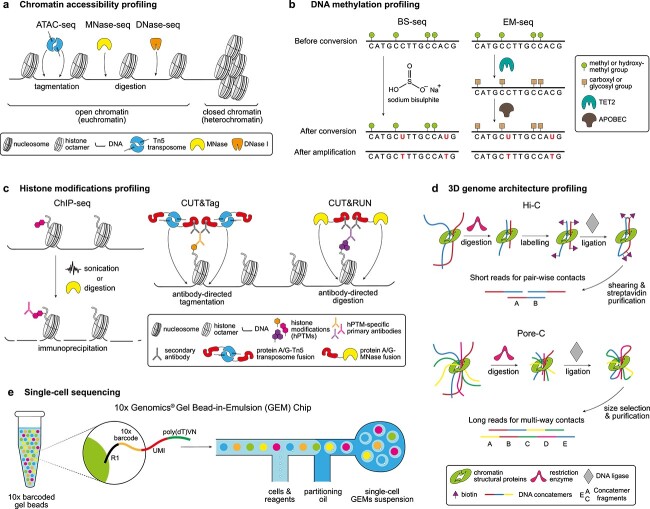
Functional genomics methodologies. (**A**) To profile chromatin accessibility, MNase-seq (centre) and DNase-seq (right) rely on the preference of DNase I and the micrococcal nuclease (MNase), respectively, for open chromatin. The more favoured ATAC-seq protocol (left) relies on a hyperactive Tn5 transposase-based transposome that has a strong bias for accessible chromatin. (**B**) DNA methylation profiling employs chemical (BS-seq, left) or enzymatic (EM-seq, right) approaches to convert unmethylated cytosines (C) into uracils (U) which, after amplification, are converted into thymines (T). This way, sequenced cytosines represent methylated (or hydroxymethylated) cytosines, whereas thymines in cytosine positions reveal unmethylated nucleotides. (**C**) ChIP-seq profiling (left) of hPTMs is based on the immunoprecipitation of sonicated or digested chromatin, often previously crosslinked. In contrast, an antibody-tethered protein A/G fused to either the hyperactive Tn5 transposase (CUT&Tag, centre) or the MNase (CUT&RUN, right) cuts chromatin only around the antibody-targeted epitope of interest, all under *in situ* conditions. (**D**) In chromosome conformation capture techniques, digested crosslinked chromatin is subjected to proximity ligation before release and purification of resulting concatemers. Hi-C (top) uses biotin-streptavidin purification of labelled fragments before shearing the DNA concatemers for short read sequencing, allowing for the identification of pair-wise contacts. The recent Nanopore sequencing-based Pore-C (bottom) lacks a labelling stage and instead purifies long DNA concatemers through a size-selection stop, thus leading to the profiling of multi-way contacts. (**E**) The 10× Genomics® GEM Chip is the most popular scRNA-seq platform. In a microfluidics platform, gel beads with barcoded primers are paired with cells coming from single-cell suspensions, and then emulsioned in a partitioning oil. The 10× barcode therefore identifies each individual cell, while the unique molecular identifier is specific to each mRNA molecule.

Gene expression regulation occurs at both the chromatin level and the post-transcriptional and post-translational one, such as in the case of RNA-mediated regulation [3]. Chromatin-level regulation is the most widely studied case, with multiple epigenetic mechanisms such as DNA methylation, histone modifications and 3D genome architecture converging and cooperating to establish complex regulatory programmes. Chromatin state and its degree of packaging are critical, as compacted heterochromatin is essentially transcriptionally inactive, whereas accessible euchromatin enhances gene expression [3, 4]. The study of chromatin accessibility through genome-wide profiling techniques, therefore, constitutes a robust proxy for understanding chromatin states and the result of epigenetic regulation. With exceptions of a few techniques like FAIRE-seq [5] that rely on crosslinking and physical shearing of chromatin, most chromatin accessibility profiling procedures, i.e. DNase-seq, MNase-seq and the more popular ATAC-seq [4–6], make use of the bias of nucleases or transposases for accessible chromatin to detect loosely packed genomic DNA (Figure 2A).

Chromatin accessibility profiling in Spiralia is unfortunately almost non-existent, with only a handful of recent studies focusing on developmental and evolutionary biology studies (Figure 1). Within molluscs, ATAC-seq profiling in the hepatopancreas of the oyster *Crassostrea gigas* [7] identified the PDX (XLOX) gene regulatory network and its regulation of insulin and insulin-related proteins as most likely ancestral to Bilateria. ATAC-seq of *Euprymna scolopes* allowed for the characterisation of microsyntenic compartments likely responsible for the evolutionary novelties in the cephalopod nervous system [8] and a study in the scallop *Mizuhopecten yessoensis* investigated the evolution of sex chromosomes by comparing ATAC-seq profiles of male and female gonads [9]. In Platyhelminthes, chromatin accessibility has been used to profile regulatory regions in stem cells and during axial patterning in the planarian *Schmidtea mediterranea* [10–12] and chromatin structure after treatment with a potential anti-helminth in *Schistosoma mansoni* [13].

Annelida is the other spiralian phylum with existing data. Our lab’s chromatin accessibility profiling of the female adult of *Dimorphilus gyrociliatus* [14] was amongst the first profiling of open regulatory regions in Spiralia. Among other findings, we identified a CTCF-binding motif as the most abundant, which we hypothesised controls gene expression regulation rather than genomic architecture, as in mammals. More comprehensive was our last study on the evolution of bilaterian life cycles [15], in which we carried out an ATAC-seq time series across five equivalent developmental stages in the annelids *Capitella teleta* and *Owenia fusiformis*. Combining these data with other functional genomics datasets, we discovered that the timing of activation of key gene regulatory networks, particularly the trunk patterning programme, correlates and likely explains the presence or absence of larval stages and the nutritional lifestyle of these larvae. Together, these early studies exploring chromatin accessibility in annelids, molluscs, and flatworms demonstrate the importance of Spiralia to understanding the evolution of Protostomia and Bilateria more broadly, as well as identifying the genomic principles underpinning key evolutionary novelties.

### DNA methylation

DNA methylation is the most abundantly studied base modification across eukaryotes [16–18]. This epigenomic mark modulates gene expression and serves various functions in biological processes. Although the predominant form, 5-methylcytosine (5mC), is primarily observed within CpG dinucleotides across eukaryotes [19–23], methylation can also occur in non-CpG contexts in plants and fungi, as well as in the brain and pluripotent stem cells in vertebrates [24, 25]. In mammals, these CpGs are dispersed in gene bodies and transposable elements (TEs) and are heavily methylated, except for the regulatory CpG-rich islands (CGI), typically located at gene promoters [22, 26, 27]. These modifications are catalysed by the DNA methyltransferases (DNMTs) [26–28], whereas methyl-CpG-binding domain (MBD) proteins recognise and bind to methylated CpG sites [29–35] and active demethylation is mediated by ten-eleven translocation (TET) methylcytosine dioxygenases, which oxidise 5mC to a 5-hydroxymethylcytosine (5hmC) state [36–40]. This DNA methylation toolkit is broadly conserved in animals and can be easily identified in available genomes and transcriptomic data. However, their function and relationship to methylation levels are still largely unclear in Spiralia.

Various tools have been crucial in directly investigating the functional consequences of DNA methylation in animal genomes. Bisulphite sequencing (BS-seq) has become the standard method for profiling DNA methylation since its inception (Figure 2B) [41]. The whole genome bisulphite sequencing (WGBS) approach now provides methylation levels across the genome and reduced representation bisulphite sequencing (RRBS) uses restriction enzymes to enrich CpG islands [42, 43]. RRBS is a cost-effective alternative, requiring lower gDNA input and fewer reads to achieve the desired sequencing depth, but it may miss certain methylation sites. Enzymatic Methyl-seq (EM-seq) (Figure 2B) was developed as an alternative, low-cost method to mitigate DNA degradation intrinsic to bisulphite treatment, which captures both 5mC and 5hmC state in a two-step reaction [44]. For non-model research systems, however, obtaining a genome-wide, single-base resolution methylation profile presents a major challenge as it is both costly and relies on the availability of a reference genome. Recently, Klughammer and colleagues [45] offered a solution by combining an optimised RRBS protocol with their RefFreeDMA software, which enabled them to survey 580 species, with a majority lacking a reference genome [46]. Nine of the species surveyed belonged to Spiralia, revealing diverse levels of global methylation. Thus, this could well be a starting point in studying this epigenomic mark in Spiralia whilst awaiting high-quality genome assemblies. Alternatively, the use of nanopore sequencing, which allows for simultaneous sequencing of 5mC methyl marks alongside the genome sequence could be employed, although it might be more costly.

Despite recent advancements, spiralian methylomes are notably scarce. Our understanding of DNA methylation in this group primarily comes from chromatography-based and methylation-specific restriction enzyme methods as well as *in silico* predictions in molluscs, with some research done in Platyhelminthes, rotifers and annelids [47–56]. Molluscs, namely *C. gigas*, have received particular attention with the salient use of BS-seq revealing that the 5mC mark is primarily restricted to gene bodies of highly expressed genes [47, 57–59]. Methylation is only moderately enriched in TEs, where intragenic and younger TEs are the primary targets [23, 58, 60]. Interestingly, studies in cephalopods demonstrated that the 5mC enrichment in TEs are disproportionately lower than the abundance of TE in the genome of *Octopus bimaculoides* [23], challenging the premise that large genome size and TE abundance are positively correlated with methylation levels [23, 61, 62]. In general, 5mC has been implicated in various biological processes across molluscs, including ageing, abiotic/biotic stress regulation with transgenerational effects, and development [47, 59].

Annelids were surveyed early on for the 5mC machinery, but research on this topic has remained scarce over the years [53–55]. Most studies have centred around the interplay between ecotoxicology and the plasticity of 5mC in earthworms [63–69]. Recently, experimental evidence in *Platynereis dumerilii* demonstrated the importance of 5mC in its regeneration capabilities [56]. *In silico* prediction using the CpG observed and expected ratio showed higher global methylation levels at the embryonic stage than reported in *C. gigas* [56, 60, 70]. Like *C. gigas*, *P. dumerilii* also experiences dynamic changes in its 5mC landscape during development and depletion in adulthood. This is not uncommon, as it has been observed in some vertebrates and invertebrates and is the result of TET-mediated demethylation [71]. It will be important to explore if this adult demethylation is conserved across Spiralia.

Methylomes of three Platyhelminthes with parasitic lifestyles exhibit interesting 5mC patterns. While it is reportedly absent in *S. mansoni,* which is likely associated with the loss of DNMT1 and DNMT3 [67], low methylation levels are observed in both *Macrostomum lignano* (adult) and *Taenia solium* (larva), consistent with the conservation of the DNMT genes in these species [72, 73]. Notably, the non-CpG is the dominant context in the genome of *T. solium*, although the methylation patterns do not vary between CpG and non-CpG contexts [73]. Additionally, the correlation between 5mC and transcriptional activity was deemed important in regulating parasitism-related genes in this parasite [73]. Further, genomic and transcriptomic evidence in rotifers reported the loss of DNMTs and TET genes, suggesting that they may not have 5mC [51, 52]. However, a recent study in *Adineta vaga* described for the first time the presence of the 4mC mark catalysed by the horizontally-transferred N4CMT bacterial enzyme in an animal [74].

In summary, the genome-wide 5mC profiles of spiralians generated to date already reveal great diversity across Spiralia. Gene body methylation is a recurrent signature in these methylomes. Still, it is unclear if the positive correlation between 5mC and gene expression observed in the oyster *C. gigas* is widespread across spiralians. Unlike in vertebrates, there is no clear targeting of TEs in Spiralia. Thus, the broader question remains about DNA methylation’s role and importance to gene regulation in this animal group. Addressing these knowledge gaps requires expanding sampling efforts across this clade, particularly by dissecting the genomic distribution of 5mC at single-base resolution.

### Histone modifications

Histone-based regulation is one of the most versatile and logically intricate mechanisms of gene expression regulation [75, 76]. Among other things, it involves the deposition and removal of posttranslational modifications, such as methyl and acetyl moieties, to key residues primarily located in the tails of histones [3, 77]. Certain histone modifications (hPTMs) cause structural perturbations in the nucleosome structure, thereby changing the level of chromatin condensation or accessibility. In contrast, others act as binding marks for modules of reader proteins and complexes that recruit chromatin-modifying machinery, leading to different biological readouts [75, 78–80]. In this way, hPTMs define regulatory regions in the genome and their levels of regulatory activity [81], and constitute critical regulators of various processes like proliferation, differentiation and development [82–84].

Research interest in histones across a wide range of spiralian species, including molluscs [85–88], annelids [89–91] and nemerteans [92], peaked during the second half of the 20th century, primarily limited to biochemical studies on differences in histone sequence, structure and composition between species. This interest soon faded, and to this day, very little is known about the impact of hPTMs on biological processes across Spiralia and whether what has been described in model organisms extends to this large animal clade. ChIP-seq, the most widely used functional genomics technique to profile hPTMs across eukaryotes (Figure 2C) [93, 94], has so far been restricted to Platyhelminthes (Figure 1), where efforts have been focused on understanding stem cell maintenance and regeneration in the planarian *S. mediterranea* [95], and the life cycle and therapeutic possibilities of the parasitic helminth *S. mansoni* [96].

In the first study utilising ChIP-seq, researchers identified distinct patterns of the transcriptionally permissive H3K4me3 mark deposited by the SET1 (SETD1A/B) and MLL1/2 (KMT2A/D) lysine-specific histone methyltransferases (KMT) orthologs in FACS-isolated *S. mediterranea* neoblasts [97]. As previously observed, H3K4me3 was positively correlated with transcription, and distinct methylation patterns (e.g. mark width) and loci were found for the two enzymes. Notably, loci required for stem cell maintenance were identified as targets of H3K4me3 methylation by SET1, whereas MLL1/2 was involved in posing ciliogenesis genes for transcription during stem cell differentiation. Later efforts [98] identified other KMTs too—the partial orthologs to MLL3 (KMT2C) and MLL4 (KMT2B)—as critical for neoblast maintenance and their differentiation into neuronal and epidermal lineages, as shown by their regeneration-deficient RNAi phenotypes.

hPTM-targeting functional genomics in planarians has also confirmed the evolutionary conservation of bivalent domains across metazoan stem cells [99]. These domains display permissive H3K4me3 and repressive H3K27me3 marks, commonly associated with transcriptionally poised genes in stem cell populations [100]. By carrying out a broad ChIP-seq profiling of four different hPTMs and the Ser5-phosphorylated RNA polymerase II (RNA Pol II), the authors confirmed the presence of these H3K4me3^+^ H3K27me3^+^ domains with a high degree of RNA Pol II pausing in planarian neoblasts. Likewise, the epigenetic regulation of oncogenes and tumour suppressor genes by MLL3/4 and SET1 has also been deemed evolutionarily conserved in stem cells, thanks to hPTM profiling in *S. mediterranea* [98, 101]. Further exploration of other hPTMs—namely H3K4me1 and H3K27ac—allowed for the annotation of putative enhancer-like elements and identification of transcription factor binding sites in enhancers [10], thus leveraging a compelling aspect of this technique: the unbiased annotation of regulatory genomic regions in non-model research systems with newly sequenced genomes.

*Schistosoma mansoni* is one of the six agents that cause schistosomiasis [96, 97], and the impact of this parasite on global health has led to a more focused interest in understanding the parasite’s life cycle and to identify therapeutic targets. These concerted efforts have led to the description of numerous hPTM profiles across the life cycle of *S. mansoni*, and even the annotation of chromatin states [102–104]. Early research identified differences in crucial hPTM levels across parasite strains that correlated with the ability to infect an intermediate mollusc host. Interestingly, the worms have also been reported to respond to introducing a new allopatric mollusc host strain with inheritable hPTM epimutations, i.e. changes in hPTM profiles [105]. This is hypothesised to explain the flatworm’s remarkable adaptability. A subsequent epigenomic profiling study used ChIP-seq to profile H3K4me3, H3K27me3, H3K9me3 and H3K9ac in the cercariae (larvae), schistosomula (post-larval specimens) and adult worms of *S. mansoni* [106]. It described the conserved punctate nature of H3K4me3, H3K9me3 and H3K9ac, as well as the characteristic broad H3K27me3 domains. More importantly, the study proved that cercariae display bivalent domains with a poised transcriptional state, which undergo repressive H3K9me3 and H3K27me3 marks loss in the schistosomula and adult stages, providing critical insights into how the parasite poises transcription until infection. A further developmental time course of H3K4me3, H3K27me3 and H4K20me1, with an even higher temporal resolution across five developmental stages (including miracidia and sporocysts), revealed the extremely dynamic nature of histone methylation across the *S. mansoni* life cycle [107]. This ultimately led to the identification of H3K27me3 methyltransferases G9a (KMT1C, EHMT2) and EZH2 (KMT6) inhibitors as development-arresting compounds and potential drugs to treat schistosomiasis.

In this same line, a vast body of research in *S. mansoni* has focused on the characterisation of inhibitors of histone-modifying enzymes [96, 108], particularly histone deacetylase (HDAC) [109], as potential drugs against the disease. ChIP-seq data have shown that treating schistosomula with the broad-spectrum HDAC inhibitor trichostatin A leads to changes in hPTM profiles in the differentially expressed genes [110]. Further, hPTM-focused functional genomics have also been used to investigate the role of hPTMs in sex-chromosome biology in *S. mansoni*, with some work done on the sex-determined regulation of gene expression [111] and gene dosage compensation in the sex chromosomes [112]. Immunologists have also shown that plasma from rhesus macaques self-cured from a schistosome infection causes epigenetic reprogramming of hPTM and gene expression in co-cultured schistosomula [113]. Lastly, other groups have used publicly available H3K4me3 and H3K27me3 ChIP-seq datasets to understand better lncRNA biology in these helminths [114–116].

Recently, new methodologies such as ChIPmentation have overcome some of the technical barriers of ChIP-seq. However, the most remarkable advancements have been made through the innovative CUT&Tag [117, 118] and CUT&RUN [119, 120] techniques (Figure 2C). These techniques are crosslinking-free and enable *in situ* profiling with an exceedingly high signal-to-noise ratio. Except for a recently published protocol for ChIPmentation in *S. mansoni* [103, 121], these novel approaches have only been successfully applied in free-living Spiralia in a single study for ChIPmentation [11] and another one for CUT&Tag [12]. The former analysed H3K27ac in dissociated cells from regenerating wounds in *S. mediterranea*, enabling the identification of regeneration-responsive enhancers and the proposition of a model to explain the chromatin accessibility rearrangements during wound healing for normal regeneration [11]. The latter study built on previous ChIP-seq research that characterised the piRNA-mediated regulation of TEs in *S. mediterranea* [122]. Through CUT&Tag profiling of H3K4me3 and H3K9me3 across cells and tissues of wild-type and *smedwi-2* (a planarian PIWI ortholog) RNAi knockdown, the authors identified SMEDWI-2 as a critical repressor of transposon misregulation in neoblasts and a chromatin chaperone during cell differentiation [12].

In summary, the wealth of data generated through ChIP-seq provides researchers in planarian regeneration and schistosomiasis with a tremendously valuable resource. Nevertheless, this must be extended to other critical spiralian phyla, such as molluscs and annelids, and refined by routinely incorporating CUT&RUN and CUT&Tag to gain a better picture of how genome regulation underpins the fascinating phenotypic diversity of Spiralia.

### 3D genome architecture

3D genome architecture describes how chromatin is spatially organised at higher levels in the nucleus, its resultant functionality and what mechanisms dictate this organisation. Two primary levels of regulation have been described thus far: compartments and topologically associated domains (TADs). Compartments refer to chromatin that shares similar accessibility and activity status. So-called A compartments denote transcriptionally active chromatin, whilst B compartments are more likely to display a heterochromatic state [123]. TADs, on the other hand, are areas of insulated self-interaction bordered by CTCF domains [124, 125]. Within vertebrates, these structures are created *via* loop extrusion. The current model for loop extrusion describes how cohesin is loaded onto the DNA and reels the strand from both directions until a CTCF domain is reached, resulting in a loop-like structure [126]. It has been well described that TAD boundaries play an important role in regulating promoter-enhancer contacts, at least in vertebrates.

Methods for identifying and understanding 3D genome architecture primarily derive from chromatin conformation capture (3C) techniques [123]. Hi-C was first developed back in 2009 and was the first method to identify pairwise interactions on a genome-wide scale [123]. Since then, many derivatives have been established, such as Micro-C, which uses a non-specific micrococcal nuclease in place of restriction enzymes; and Pore-C, which creates long reads of multiway concatemers, thus identifying multiway interactions as opposed to pairwise ones (Figure 2D) [127, 128]. In addition to 3C-derived methods, techniques such as SPRITE, which are ligation-free, have recently been developed [129] to study 3D genome architecture.

Compartmental domains appear to be involved in genome organisation across eukaryotes, but the extent to which TADs bordered by CTCF are used across Metazoa appears to vary. TADs play an important and extended role in jawed vertebrates, yet this fact does not necessarily extend across Bilateria. For example, Ecdysozoa, the sister clade to Spiralia, shows significant variation. *Drosophila* and *Anopheles*, in addition to a CTCF, have insect-specific architectural proteins such as BEAF-32, ZIPIC and the Elba complex components [130]. Despite this, most of their organisation relies on A/B compartmentalisation, with CTCF use restricted to just a handful of loci like the *Hox* cluster [131–133], suggesting innovation and novelty in this lineage. Yet while CTCF is present in ‘basal’ nematodes—and therefore present in the last common ancestor of these—it has been secondarily lost in some derived Nematoda species like *Caenorhabditis elegans* [134]. Therefore, although CTCF was present in the last common bilaterian ancestor, its use varies significantly, even within phyla. Indeed, there is also conflicting data regarding how conserved TADs are. Early studies found strong TAD conservation in vertebrates, yet a later study suggested only a 43% TAD conservation between humans and chimpanzees [135–137]. Within drosophilids, one study concluded that 3D genome structure had largely been conserved for 40 million years, whilst another comparing *D. melanogaster* and *D. triauria* found most TADs had been largely reorganised [138, 139]. As a result, it is not well understood the extent to which TADs are conserved, likely due to variations in analytical choices and experimental design [140].

Within Spiralia, there are very pronounced knowledge gaps regarding 3D genome architecture. Very few studies are available, and the ones available cover only a tiny fraction of this highly diverse superclade. Some conservation is expected, since multiple CTCF orthologs have been identified, including three in molluscs, four in annelids and one in rotifers, suggesting the molecular machinery for TADs formed by cohesin loops is available [141]. However, some phyla, such as Platyhelminthes, appear to lack CTCF altogether, which could imply a secondary loss, as seen in nematodes [141]. The only in-depth study regarding 3D genome architecture was in the cephalopod *E. scolopes* (Figure 1), which led to the first and only description of TADs in a spiralian species. Researchers identified TAD structures and inferred that TAD formation *via* loop extrusion was the most likely mechanism behind this genomic architecture due to boundaries being enriched for a CCCTC-like motif and critical proteins involved in loop extrusion. However, the lack of identified CTCF motif directionality prevented inferring the mechanisms behind this TAD formation. The squid’s TADs were also larger than mammalian TADs, but had a much larger distribution of TAD sizes [8], revealing novel insights into 3D genome architecture that had not been described before in model systems.

Despite 3D genome architecture being an increasingly popular topic in molecular and cell biology, the lack of interest in Spiralia is overwhelming. Our understanding of this process in metazoans definitely requires the study of spiralian species because current studies cover only a fraction of Bilateria and, in some cases, show conflicting information. Although it appears the machinery for TADs via loop extrusion could predate bilaterians, not all animals utilise this method as their primary tool for genome organisation, and some lack CTCF altogether. Studies in Ecdysozoa have shown that closely phylogenetically related animals can show significant differences in organisation, highlighting that the presence of TADs in *E. scolopes,* coupled with the lack of CTCF in Platyhelminthes, could show a similar level of diversity within Spiralia.

### Single-cell sequencing

Since the advent of single-cell sequencing in 2009 [142], technologies have advanced considerably [143], elucidating the heterogeneity of cell populations in terms of gene expression, epigenetic modifications [144, 145], chromatin accessibility [146], cell surface proteins [147] and their spatial distributions within tissues [148]. Although combining single-cell sequencing with other profiling techniques is increasingly popular, the most commonly used single-cell technology is the traditional single-cell RNA-seq (scRNA-seq) (Figure 2E) [149].

While the methods for performing scRNA-seq are increasingly used on various sample types, Spiralians have yet to be profiled to any great extent with these methods. This is partly due to some spiralians presenting problems in dissociation steps. Molluscs, for example, contain many adhesive structures and mucus, which contains polyphenolic proteins and mucopolysaccharides that can inhibit enzyme-catalysed molecular procedures, or affect dissociation success [150]. Despite this, some progress has been made in Spiralia, although only molluscs, annelids and Platyhelminthes have any published literature in which scRNA-seq has been utilised. The species explored across these papers include the five molluscs *Dreissena rostriformis, B. glabrata, M. yessoensis, C. gigas* and *Crassostrea hongkongensis* [151–156], the four annelids *Eisenia andrei, P. dumerilii*, *Pristina leidyi* and *C. teleta* [157–161], and three platyhelminthes, *S. mediterranea, Prosthecereaus crozieri* and *S. mansoni* [162–167]. Though better than for the study of other aspects of genome regulation, this is a poor sample of the observed diversity in Spiralia, with only three out of the 15 constituent phyla of Spiralia having been explored and tested through these methods (Figure 1).

In molluscs, comparative single-cell transcriptomics has been used to examine the formation of both embryonic and larval shells in the quagga mussel *D. rostriformis.* This revealed significant differences in gene expression between the two stages, while also determining that the biomineralisation toolbox utilised in shell formation, co-opted by molluscs, was already present in the last common metazoan ancestor [151]. Other studies on molluscs have investigated the developmental trajectories of cells to organs in *D. rostriformis* [152], the adductor muscle cell type diversity in *M. yessoensis* [153], larval structural homologies between larvae of *C. gigas* and the platyhelminth *P. crozieri* [167], the role of immune cell subtypes in parasitic infections in *B. glabrata* [156], and examined transcriptional changes of immune cell populations following exposure to copper contamination in *Cohnella hongkongensis* [154, 155].

For annelids, single-cell transcriptomic profiling of *P. dumerilii* has helped reveal new details about the subdivisions of the annelid body plan [158] and the complexity of the brain and nervous system [157]. scRNA-seq has also been used to profile gene families and TEs involved in the regeneration of the earthworm *E. andrei* [160]*,* profile cell types, particularly of stem cell populations, in *P. leidyi* [161]*,* and conduct pseudo-time analysis on neurosecretory clusters of cells in *C. teleta* to reveal temporally distinct cell states and discover two potentially distinct neural differentiation trajectories [159].

In Platyhelminthes, single-cell technologies have been used in several ways, mainly to produce large cell atlases for *S. mediterranea*, which have revealed transient regeneration-activated cell states (TRACS) in multiple tissues, including muscle, epidermis and the intestine; and when genes found enriched in these cells are depleted using RNAi, regeneration is incomplete [166]. Other studies in *S. mediterranea* have focused on examining the heterogeneity in the planarian neoblasts [164], profiling cell types [162, 168], and examining the extent to which specialised cell types make use of both energy and biosynthesis pathways [163]. The parasitic platyhelminth species *S. mansoni* has also received some research attention, having been characterised using single-cell sequencing as both whole dissociated animals and in terms of specific tissues at various life cycle stages to help identify cell developmental trajectories [169, 170], genes underlying feeding behaviours and parasitism [171, 172] and assess its development more generally [165, 173].

Comparing the studies conducted in spiralians with non-spiralian clades reveals a major disparity in the volume of work. Many more non-spiralian animal species have been profiled from much-varied groups compared with spiralians, including almost all chordate main clades, and species from other groups such as Cnidaria, Arthropoda, Nematoda, Ambulacraria, Porifera, Ctenophora, Placozoa and Xenacoelomorpha [174–185]. Moreover, the scale of experiments in spiralians lags compared with other systems. The species in Spiralia with more single-cell work is *S. mediterranea,* an established model organism for studying whole-body regeneration. More species need to be profiled to capture cell type and expression diversity more accurately, and expand our collective understanding of processes undertaken in spiralians, like regeneration and metamorphosis. To do so, high-quality genomes of more Spiralians are paramount. Still, for those already sequenced, it will be essential to continue profiling the diversity of cell types in adults and during development to act as valuable starting points for comparative transcriptomics in other Spiralians. Most importantly, phyla aside from Annelida, Mollusca and Platyhelminthes must also be explored at the single cell level.

## Abbreviations

4mC, *N*^4^-methylcytosine; 5mC, 5-methylcytosine; 6 mA, *N*^6^-methyladenine; ATAC-seq, assay for transposase-accessible chromatin with sequencing; BEAF-32, boundary element-associated factor of 32 kDa; BS, bisulphite sequencing; CGI, CpG-rich islands; ChIP-seq, chromatin immunoprecipitation followed by sequencing; CTCF, CCCTC-binding factor; CUT&RUN, cleavage under targets and release using nuclease; CUT&Tag, cleavage under targets and tagmentation; DNase-seq, DNase I hypersensitive sites sequencing; DNMT, DNA methyltransferase; dsRNA, double-stranded RNA; EHMT2, euchromatic histone-lysine *N*-methyltransferase 2; Elba complex, Early boundary activity complex; EM-seq, enzymatic methyl-seq; EZH2, enhancer-of-zeste homolog 2; FACS, fluorescence-activated cell sorting; FAIRE-seq, formaldehyde-assisted isolation of regulatory elements; hPTM, histone post-translational modification; H3K4me1, monomethylation of lysine 4 of histone H3; H3K4me3, trimethylation of lysine 4 of histone H3; H3K9ac, acetylation of lysine 9 of histone H3; H3K9me3, trimethylation of lysine 9 of histone H3; H3K27ac, acetylation of lysine 27 of histone H3; H3K27me3, trimethylation of lysine 27 of histone H3; KMT, lysine-specific histone methyltransferase; lncRNA, long non-coding RNA; MBD, Methyl-CpG-binding domain; MLL, myeloid/lymphoid leukaemia (also: mixed-lineage leukaemia); MNase-seq, micrococcal nuclease digestion with deep sequencing; PDX, pancreatic and duodenal homeobox; piRNA, piwi-interacting RNA; PIWI, P-element Induced WImpy testis; RNAi, RNA interference; RNA Pol II, RNA polymerase II; RRBS, reduced representation bisulphite sequencing; scRNA-seq, single cell RNA-seq; SET, su(var)3–9 enhancer-of-zeste and trithorax; SETD, SET domain-containing; SPRITE, Split-pool recognition of interactions by tag extension; TAD, topologically associated domain; TET, ten eleven translocase; TRACS, transient regeneration activated cell states; UMI, unique molecular identifier; XLOX, *Xenopus laevis* homeobox; ZIPIC, Zinc-finger protein interacting with CP190

**Key Points**

- Our understanding of functional genomics in Spiralia is limited, although this is one of the three main lineages of bilaterally symmetrical animals with many economic and biomedical significant species.
- We need a better understanding of genome regulation in spiralians, with histone modification profiling restricted to, e.g. ChIP-seq analysis in two flatworm species.
- Methylation levels vary in Spiralia, but the low number of available methylomes prevents dissecting the evolutionary history of this epigenetic mark and its impact on gene expression regulation in Spiralia.
- Further work in 3D genome architecture in multiple spiralian phyla is needed to conclude high-order genome regulation in this group and animals in general.
- scRNA-seq has provided critical insights into regeneration, immune cell diversity and stem cell heterogeneity in a handful of phyla in Spiralia, but trialling other groups is needed for a complete understanding of spiralian biology.

## Notes

**Francisco M. Martín-Zamora, Billie E. Davies, Rory D. Donnellan, and Kero Guynes** are early career researchers doing their PhD projects in the Martín-Durán lab, led by **José M. Martín-Durán**, at Queen Mary University of London.
